# Opioid use, regulation, and harms in Brazil: a comprehensive narrative overview of available data and indicators

**DOI:** 10.1186/s13011-021-00348-z

**Published:** 2021-01-26

**Authors:** Lucas O. Maia, Dimitri Daldegan-Bueno, Benedikt Fischer

**Affiliations:** 1 Centre for Applied Research in Mental Health and Addiction, SFU Faculty of Health Sciences, Simon Fraser University, 515 W. Hastings Street, V6B 5K3 Vancouver, BC Canada; 2 Schools of Population Health and Pharmacy, Faculty of Medical and Health Sciences, University of Auckland, 85 Park Rd, 1023 Grafton, Auckland New Zealand; 3 Department of Psychiatry, University of Toronto, 250 College Street, 8th floor, M5T 1R8 Toronto, ON Canada; 4 Department of Psychiatry, Federal University of São Paulo, R. Dr. Ovídio Pires de Campos, 785, 05403-903 São Paulo, Brazil

**Keywords:** Opioids, Harms, Pain, Non‐medical use, Policy, Public health, Regulation, Brazil, Latin America

## Abstract

**Background:**

Global opioid consumption increased multifold post-2000, disproportionately in high-income countries, with severe mortality/morbidity consequences. Latin America features comparatively low opioid availability; Brazil, the region’s most populous country, makes an interesting case study concerning opioid use/harms. In this comprehensive overview, we aimed to identify and summarize medical and non-medical data and indicators of opioid availability and use, regulation/control, and harm outcomes in Brazil since 2000.

**Methods:**

We searched multiple scientific databases to identify relevant publications and conducted additional ‘grey’ literature searches to identify other pertinent information.

**Results:**

Despite some essential indicators, opioid-related data are limited for Brazil. Data indicate that population-level availability of prescription opioids represents only a small fraction of use in comparison to high-income countries. However, within Latin America, Brazil ranks mid-level for opioid consumption, indicating relatively moderate consumption compared to neighboring jurisdictions. Brazil has implemented restrictive regulations to opioid prescribing and is considered ‘highly restricted’ for opioid access. Codeine remains the major opioid analgesic utilized, but stronger opioids such as oxycodone are becoming more common. Professional knowledge regarding medical opioid use and effects appears limited. National surveys indicate increases in non-medical use of prescription opioids, albeit lower than observed in North America, while illicit opioids (e.g., heroin) are highly uncommon.

**Conclusions:**

Overall population-level opioid availability and corresponding levels of opioid-related harms in Brazil remain substantially lower than rates reported for North America. However, the available surveillance and analytical data on opioid use, policy/practice, and harms in Brazil are limited and insufficient. Since existing and acute (e.g., pain-related) needs for improved opioid utilization and practice appear to be substantiated, improved indicators for and understanding of opioid use, practice, and harms in Brazil are required.

## Introduction

The worldwide use of opioids has substantially increased post-2000. Based on International Narcotics Control Board (INCB) data, global consumption of opioid analgesics (in defined daily doses for statistical purposes [S-DDD] per million inhabitants per day) has risen by 250 %, from approximately 5 million S-DDD in 2000 to approximately 13 million S-DDD in 2014, yet has plateaued since [1]. The global prevalence of non-medical opioid use among persons aged 15–64 was estimated to have increased from 0.7 % (approximately 35 million people) in 2015 to 1.2 % (approximately 58 million) in 2018 [2, 3]. The majority (80–90 %) of global opioid consumption, and recent related increases, have been concentrated in high-income regions of North America, Western/Central Europe, and Oceania [1, 4]. Globally, a total of 109,500 opioid-related deaths were estimated for 2019 [5].

The highest consumption of opioids occurs in North America (e.g., the United States and Canada), where opioid availability exponentially increased, reaching the highest levels globally (~ 31,000 S-DDD) by the period 2011-13 [4, 5]. More recently – following the implementation of system-level restrictions (e.g., opioid restrictions, prescription monitoring and guidelines, enforcement) – opioid consumption levels have inverted and decreased by 20–50 % in North America from peak levels until 2018 [6–8]. Yet, in 2016-18, North America alone still accounted for about 60 % of the world’s total opioid consumption [1]. Post-2000, and fueled by the persistently high opioid availability described, North America has experienced steep increases in population-level opioid-related harms, including opioid-related morbidity and mortality (e.g., acute poisoning deaths) [9–11]. For example, a total of 47,600 opioid-related deaths occurred in the United States in 2018, with proportionally similar rates, and a total of 4,398 opioid-related deaths in Canada [12–14]. However, following policy restrictions and availability reductions, mortality resulting from prescribed opioids has steadily decreased. Additionally, there have been recent increases (60–80 %) in illicit/synthetic opioid use (e.g., fentanyl, heroin) over time, substantially driving opioid-related mortality [10, 15, 16]. In North America, high levels of opioid-related deaths negatively impact life expectancy in the general population [17–20].

While comparative data are scarce, the context of opioid use and harms in Latin America differs from that in North America [21]. In Latin America, there are significantly lower amounts of medically prescribed opioids, especially when compared to high-income countries [4]. Medical opioid consumption in Central/South America from 2016 to 18 (S-DDD/1,000,000/day) amounted to approximately 2 % of the world’s total, or < 5 % that of North America and < 12 % that of Europe [1]. Some Latin American countries only now report the levels of opioid use (about 20 mg morphine equivalents/capita) found in North America decades ago [22]. This is despite the fact that chronic pain – the main indication for opioid medications use – is highly prevalent in Latin America, with mean estimates ranging from 26 to 37 % [23, 24]. However, access to effective pain treatment involving opioid pharmacotherapy is limited or insufficient in most Latin American countries [23, 25–27]. Similarly, the prevalence of non-medical opioid use in South America was estimated to be 0.1–0.2 % from 2015 to 2017, in comparison to approximately 4 % reported in North America [2, 28].

In the specific Latin American context, Brazil features a relatively low prevalence of opioid use and harms, especially when compared to high-prevalence settings like North America. However, this situation does not compromise the need and utility of comprehensively examining related indicators, especially in the spirit of comparative study. Rather, and given that opioids offer both medical (e.g., pharmacotherapeutic) value, for example for pain treatment, as well as are the causes of extensive (e.g., mortality/morbidity) harm, and no generally accepted ‘optimum’ use levels exist, it is equally worthwhile to study ‘low-use/harm’ jurisdictions as it is to study others, including the possibility to ascertain system or practice barriers to use, determinants of lower harms, and so forth. In this particular context, Brazil represents the most populous country while still being representative of the opioid context in Latin America, and so overall offers a unique case study in regard to opioid use, harms, and regulations [4, 23]. Furthermore, Brazil features distinctly high levels of other psychoactive substance use (e.g., problematic drinking, psychostimulant, or benzodiazepines use) [29, 30]. On this basis, we aimed to identify and summarize available indicators and data on (medical and non-medical) opioid availability and use, control/regulations, harms (e.g., morbidity or mortality outcomes), and associated factors in Brazil since 2000.

## Methods

In the context of limited availability of (especially peer-reviewed journal based) data but with the aim to comprehensively consider and present information on opioid use, availability, harms, and regulations/policy in Brazil, we relied on a combination of search strategies and approaches to identify relevant indicator data and information. First, we developed basic search terms (e.g., “opioid(s) or opiate(s) or morphine or heroin” and “Brazil or Latin America,” combined with subtopic-specific additional terms, e.g., use, mortality, morbidity, pain, policy, control) and electronically searched main scientific databases, including MEDLINE (PubMed), Web of Science, Scopus, Embase, CINAHL (EBSCO), LILACS, and SciELO for relevant publications. The focus was on original studies or other publications, in English or Portuguese, originating from Brazil that included relevant data towards the scope of the present overview as defined, for the time period from 2000 to 2020. Potentially relevant publications identified were title- and abstract-screened and selected for inclusion/exclusion principally by the first author (LOM), with consultation with the co-authors in case of ambiguity. Second, we manually scanned related bibliographies and conducted Internet-based searches (e.g., by Google Scholar, Google) for additional studies and indicator data, including ‘grey literature’ (e.g., surveys, databases, organizational or technical or mass media reports) pertaining to the topic of interest. All data and indicator materials of relevance were extracted, thematically and topically structured and organized, and subsequently narratively summarized and presented. Given the specific and combined data search and identification approach, the present overview is non-systematic while comprehensive and narrative in nature; hence, also no formal review reporting system (e.g., PRISMA) or related data is presented.

## Results

### Medical opioid use in Brazil

#### Data from national/international databases

The INCB data indicate that the national level of opioid consumption (moving averages for three-year periods) in Brazil increased from 172 S-DDD/1,000,000/day (2000-02) to 384 S-DDD/1,000,000/day (2009-11) and to 512 S-DDD/1,000,000/day (2016-18) [1, 31, 32]. While these indicator data represent substantial proportional increases, opioid consumption remains significantly smaller (< 5 %) in Brazil than those of the G-20 countries. Within Latin America, Brazil’s opioid consumption, comparatively, ranks mid-level, with some countries having higher consumptions levels (e.g., Chile: 1,363 S-DDD/1,000,000/day; Argentina: 756 S-DDD/1,000,000/day) and lower consumption levels (Peru: 189 S-DDD/1,000,000/day; Bolivia: 53 S-DDD/1,000,000/day) from 2016 to 2018 [1]. These intraregional indications for population-level opioid use in Latin America are reflected by other data. For instance, the highest opioid availability (in log-distributed opioid morphine equivalents) in 2014 was reported in Argentina (34 mg/capita), followed by Chile (14 mg/capita), Panama and Brazil (about 10 mg/capita each), while Costa Rica, Peru, Mexico, and Bolivia reported levels ≤ 5 mg/capita in the same year [22].

Longitudinal data from insurance claims showed that 2.2 % of Brazilian patients of any condition (*n* = 1,057,033) and 24.4 % of cancer patients (*n* = 9,873) covered by private health care plans received opioid therapy between 2004 and 2007 [33]. A study using the National System for the Management of Controlled Substances [*Sistema Nacional de Gerenciamento de Produtos Controlados*] found that the largest portion of opioid prescriptions by Brazilian dentists dispensed in 2012 (*n* = 141,161 prescriptions) was for codeine combination (i.e., together with paracetamol or other non-opioid analgesics) and singular formulations (86.7 %), followed by tramadol only/combined (12.6 %), oxycodone (0.3 %), morphine, fentanyl, and hydromorphone (< 0.001 % each). Most prescriptions dispensed corresponded to a short period of opioid-based treatment of up to four days (four DDD by prescription, 62 %) and a maximum of a single drug package (90 %) [34]. Another study using the same database showed a 465 % increase in the number of codeine, fentanyl, and oxycodone prescriptions dispensed by Brazilian dentists from 2009 (1.6 million) to 2015 (9 million), with codeine representing > 98 % of the total prescriptions and representing the largest rate increase (8.2 to 43.4 prescriptions/1,000 people); oxycodone had the largest relative increase, rising more than ten-fold (0.07 to 0.8 prescriptions/1,000 people), while fentanyl had the smallest absolute and relative increase (from 0.02 to 0.05 prescriptions/1,000 people) [35].

#### Cross‐sectional survey data

Opioid analgesics use was reported by 2.2 % and 2.6 % of chronic pain participants from two population-based household surveys conducted in the Brazilian municipalities of São Luís/MA (2009-10; *n* = 1,597) and Botucatu/SP (2016; *n* = 416), respectively [36, 37]. Studies surveying specific disease-related populations reported comparatively higher prevalence of opioid use. A study with 280 outpatient cancer treatment patients of an oncology hospital in Curitiba/PR found that 30 % and 16 % of patients received weak or strong opioids for pain relief in 2015, respectively [38].

Among 307 neuropathic pain patients treated in three general hospitals/pain clinics based in the Brazilian municipalities of Santo André/SP or Salvador/BA, the prevalence of opioid-therapy within the last six months was 39 % (median: 32.8 %, range: 0.0 %-39.3 %), post-surgical neuropathic pain (39 %) and chronic lower back pain with a neuropathic component (34 %) as the most frequent conditions for which opioid prescriptions were issued [39].

In a nationally representative household survey (*n* = 41,433) conducted in 2013-14, 0.5 % of respondents reported opioid analgesics use to treat pain associated with chronic diseases (current continuous use: 29 % of those who had reported use) or acute diseases/events (occasionally within the last 15 days: 71 %). Among respondents indicating any medical analgesic use (*n* = 13,054), 1.7 % used opioids, of whom 39 % used codeine, 31 % papaverine, and 26 % tramadol. Opioids were predominantly used in combination formulations (e.g., codeine-paracetamol), including non-opioid analgesics (e.g., paracetamol, metamizole) (1.7 %), non-steroidal anti-inflammatory drugs (NSAIDs 0.4 %), or both (0.4 %). The prevalence of medical opioid use in the general population was significantly higher among people aged 60 years or older (0.8 %) compared to the 20–59 years-of-age group (0.5 %) [40].

### Contextual and regulatory factors related to medical opioid use in Brazil

#### Regulation of prescription opioids

The Brazilian National Health Surveillance Agency (ANVISA) is part of the Brazilian National Health System, in charge of protection and regulation (e.g., approvals) of health products and services, including prescription medications/opioids. Opioid analgesics are scheduled as “narcotic substances” (List “A1”, including morphine, buprenorphine, pethidine, methadone, hydrocodone, oxycodone, fentanyl, and others) or “narcotics of which use is permitted only in special concentrations” (List “A2”, including codeine, tramadol, dextropropoxyphene, nalbuphine, and others), following the general division into ‘strong opioids’ and ‘weak opioids’ from the WHO’s ‘pain ladder’ [41]. Only medical physicians and dental surgeons are permitted to prescribe scheduled opioids, based on a special registration from the local health surveillance service. Further, pharmacists are not allowed to accept emergency telephone prescriptions for opioids or to correct technical errors (e.g., misspelling, missing values) on a prescription in order to dispense the medication [25, 42]. In addition to a duplicated special prescription form, prescribers need to provide a document termed “Prescription Notification” [*Notificação de Receita*] containing the prescriber, patient, and provider identifications; each form covers up to a maximum of 30 days of prescription supply for treatment. A prescriber’s written justification is required for longer periods. Dispensing entities (i.e., pharmacies, drugstores) must register dispensing through the National System for the Management of Controlled Substances [*Sistema Nacional de Gerenciamento de Produtos Controlados*] run by ANVISA and forward all Prescription Notifications monthly to local Health Authorities, which retains one copy and returns the other to the dispensing entity after verification [42, 43]. Currently, opioids scheduled and available for outpatient use in Brazil include codeine, morphine, tramadol, methadone, buprenorphine, oxycodone, and fentanyl [44]. Codeine and morphine are included in the national list of essential medicines used within the public health system and, so consequently, make up the majority of opioid prescriptions dispensed [45].

#### Medical and patient education

A survey in Porto Alegre/RS (2011-12) involving 122 physicians, pharmacists, physiotherapists, nurses, and nursing technicians/assistants working in oncology and intensive care pediatric units from a general hospital reported that half (51 %) had no prior pain management training; 82 % were unclear about or confused opioid-related withdrawal, tolerance, and dependence symptoms; 20 % believed that patients’ asking for higher opioid doses is indicative of addiction; 42 % believed opioid-related respiratory depression to be common; 47 % assumed that opioids ought not to be used upon unknown causes of pain [46]. Among 126 nurses at an oncology center in Rio de Janeiro/RJ, half (48 %) believed that opioids harm patients, while the belief that opioids do not cause harm (52 %) was associated with adequate knowledge on cancer pain management [47]. Among 257 opioid-prescribing dental surgeons from the state of Minas Gerais, almost two-thirds (62 %) reported lack of knowledge of Brazilian opioid-related regulatory legislation, and legislation knowledge was associated with higher prescription frequency [48].

A prospective study (2005 to 2009) in a São Paulo/SP private hospital evaluated pethidine and morphine prescription amounts after the implementation of an educational protocol informing prescribing physicians about toxicity of pethidine and suggesting its replacement by morphine. Results found a significant decrease (72 %) in pethidine prescriptions (in milligrams/year), as well as a significant increase (42 %) in morphine prescriptions over the study period [49].

A patient-focused study conducted in an oncology hospital in Curitiba/PR showed that only 41 of 280 (15 %) cancer patients correctly classified morphine as an opioid analgesic, while 19 % would refuse taking morphine even if prescribed by their doctors due to fear of addiction (65 %), tolerance (30 %), or adverse reactions (35 %), 68 % believed that opioid use is directly related to worsening disease outcomes and 41 % that opioid use means that death is closer [38].

#### Socioeconomic factors

The mean expenditure by patients on a single opioid prescription was calculated to be 5 % (Brazilian Reais [R$]33.27) of the Brazilian minimum wage (R$678.00) in 2013, with codeine being the lowest (R$29.59) and oxycodone the highest (R$300.08 ) mean cost per prescription [50]. Accordingly, high-income states showed higher opioid dispensing rates, with 87 % of all prescriptions dispensed in 2012, mostly concentrated in South and Southeast Brazilian states. These variations significantly correlated with socioeconomic status (e.g., poverty, human development index, education) and health-related (e.g., prescriber access) indicators of the corresponding jurisdictions [34].

### Non‐medical opioid use

#### Prescription opioids

Results from national household surveys (2005 and 2015) suggest that among Brazilians aged 12–65 years there was an increase in non-medical opioid analgesic use, defined as non-prescribed use. Corresponding rates increased from 1.9–2.9 % for lifetime use, 0.5–1.4 % for past-year use, and 0.3–0.6 % for use in the past-month, although these differences have not been assessed statistically [28, 51, 52]. In 2015, the annual prevalence of non-medical opioid use (1.4 %) was substantially lower than the corresponding use rates for alcohol (43 %) or tobacco (17 %), but similar to the use prevalence of cannabis (2.5 %), benzodiazepine (1.4 %), or cocaine (0.9 %) and amphetamine (0.3 %)  [51]. The majority of individuals who have used opioids in the past-month reported limited use; for instance, persons would use infrequently – 1–2 days per month (35 %) or 3–5 days per month (27 %) [28]. Women’s non-medical opioid use was higher than that of men both in 2005 (1.6 % vs. 0.9 %) and 2015 (1.8 % vs. 1.0 %) [28]. In addition, the 2015 national survey found that male sex (prevalence ratio: 0.53 [0.36–0.78]), younger age (10–24 years compared to 45–65) (PR: 0.56 [0.34–0.92]), monthly family income of R$1,501-3,000 (PR: 0.59 [0.38–0.92]) or greater than R$3,000 (PR: 0.64[0.42–0.98]) compared to the lowest income group (up to R$750), and being unemployed (PR: 0.65 [0.46–0.92]) were all significantly associated (*p* < 0.05) with a lower prevalence of non-medical opioid use. Conversely, there were no significant differences in use prevalence between different ethnic/racial groups, education levels, or religions (*p* > 0.05) [28].

A national survey among college students from Brazilian state capital cities (2009) reported prevalence of non-medical (non-prescribed) opioid use, with findings as follows: 5.5 % (lifetime use), 3.8 % (use in the past-year), and 2 % (use in the past-month). When comparing results by sex, women showed significantly higher prevalence of lifetime use (6.3 % vs. 4.4 %), past-year use (4.8 % vs. 2.2 %), and past-month use (2.7 % vs. 1.0 %) of opioids non-medically, and higher prevalence (1.3 % vs. 0.4 %) of hazardous opioid use (i.e., “moderate risk”) as assessed by the World Health Organization Alcohol, Smoking, and Substance Involvement Screening Test (ASSIST-WHO) [53]. In addition, a separate national survey conducted among high-school students in Brazilian state capital cities estimated the prevalence of lifetime non-medical (non-prescribed) opioid use at 0.3 % in 2004 and 0.6 % in 2010, without statistical difference between men and women [54, 55].

When considering opioid dependence (DSM-IV criteria), the 2015 national household survey reported a 0.1 % (or about 208,000 people) prevalence among people aged 12–65 years. These estimates were higher (not statistically tested) among respondents aged 25–34 years (0.3 %) and 35–44 (0.2 %), and among women (0.2 %), as compared to men (< 0.1 %) [51]. High levels of opioid involvement were reported by a survey of physicians (*n* = 198) receiving outpatient service treatment for substance dependence across Brazil (2000 to 2005). Prescription opioids were the second-most common substance abused (23 %), following alcohol (49 %) but comparable to cocaine (21 %) [56]. Among a sample of anesthesiologists (*n* = 57) who sought outpatient treatment for substance use disorders in São Paulo between 2002 and 2009, opioid abuse was most commonly reported by 34 (60 %) respondents, and almost all of which (88 %) showed consumption patterns consistent with dependence symptoms [57]. It was estimated that about 30,000 Brazilians aged 12–65 years were receiving treatment (mainly psychosocial or psychiatric) for opioid-related use problems in 2015 [51].

#### Heroin

Other than in North or Central American regions, the use of heroin or other illicit opioids is uncommon in Brazil, and data are correspondingly scarce. National household surveys have estimated the prevalence of lifetime heroin use of 0.1 % in both 2001 and 2005 [52, 58], 0.2 % in 2012 [59], and 0.3 % in 2015 [28, 51]. The prevalence of past-year use was estimated at < 0.1 % in 2005 and 2015 [28, 52]. In a national survey conducted across Brazilian state capital cities (2003) examining street-involved/homeless youth (9–18 years), 122 of 2,807 (4.3 %) respondents reported lifetime injecting drug use; however, the type of drugs used was not specified. The study highlighted the use of psychostimulants without explicitly noting heroin [60]. Similarly, the use of non-specified injecting drug use was reported by 10 of 330 (< 3 %) homeless people from São Paulo [61].

Despite the scarcity of scientific data, select media reports have described sporadic occurrences of heroin distribution and usage in local areas of São Paulo, Brazil’s largest urban center characterized by illegal drug use (‘cracolandias’) in which psychostimulants (e.g., crack-cocaine), alcohol, and cannabis are the predominant drugs of consumption [62]. Heroin use in these sub-locales occurs mostly by inhalation and smoking (similar to the use of crack-cocaine) instead of injecting. However, with the high cost and limited access of heroin, this practice is uncommon [63, 64]. Media reports and police investigations indicate sporadic heroin use in settings like São Paulo. These occurrences are irregular and are thought to be mostly from West African importation and then distributed to end-users by African migrants and asylum seekers [64]. Other select heroin seizures have been reported in the media: in 2003, 15 kg of heroin from Colombia was seized by the Federal Police in the North region (Amapá State), adding up to almost 100 kg seized between 2001 and 2003 [63]. Further, in 2018, Brazilian customs seized 100 kg of heroin at Rio de Janeiro/RJ airport, imported from Hong Kong concealed as a licit chemical (fluticasone) for medication production by a Brazilian firm [65]. Otherwise, there are virtually no indications of recurring opioid use in Brazilian drug use scenes.

#### Opioid‐related morbidity/mortality

In 2007, there were 138,585 hospitalizations recorded by the Brazilian public health system (through the Hospital Information System *SIH-SUS*) as ‘mental and behavioral disorders due to psychoactive substance use.’ Of these, 2,232 (1.6 %) were associated with opioid-related disorders, although the reason for hospitalization (e.g., acute intoxication, physical or psychological complications from harmful use, dependence/craving symptoms) was not reported. This rate is substantially lower than for alcohol (69 %), multiple drugs (23 %), or cocaine (5 %) but higher than for cannabinoids (0.8 %) or sedatives/hypnotics (0.5 %) [66].

Similarly, mortality data are scarce and limited in specificity. Data from the Mortality Information System (*SIM*), run by the Health Surveillance Secretariat of Ministry of Health, shows 44,326 deaths associated with psychoactive substance-related disorders (reason unspecified) from 2001 to 2007, with 24 (0.1 %) related to opioids; a substantially lower proportion than alcohol (86.6 %), tobacco (6.3 %), multiple drugs (0.7 %), or cocaine (0.4 %), but similar to cannabinoids (0.1 %), sedatives/hypnotics (0.1 %), or inhalants (i.e., solvents) (0.1 %) [66]. For the period 1998 to 2018, 111 deaths (0.08 %) out of all psychoactive substance-related deaths (*n* = 141,218) were reported as associated with opioids, of which 72 (65 %) and 39 (35 %) involved male and female decedents, respectively [67]. From 2010 to 2015, 2642 (67 %) poisoning deaths (i.e., accidental, intentional (suicide), or undetermined intent) were associated with ‘narcotics and psychodysleptics [hallucinogens]’, and 1060 (27 %) were associated with acute alcohol intoxication (*n* = 3,927) [68]. However, these general and unspecific substance-categorizations render it difficult to ascertain the actual number of opioid-related poisoning deaths in Brazil.

## Discussion

Available indicators document that population-level availability and use of prescription opioids in Brazil constitutes a small fraction compared to that of high-income countries, especially in North America (i.e., the United States, Canada), which is home to the world’s highest opioid consumption levels and related adverse outcomes (e.g., mortality, morbidity). In essential ways, this contrast in opioid use within the Americas region is an apt exemplification of the extreme differences in opioid consumption between wealthy and poor/threshold countries. As is true for many other countries, opioid consumption in Brazil has increased – by about 200 % S-DDD/1,000,000 population/day – since 2000, however, at comparably low levels. Within Latin America, Brazil ranks in mid-field, indicating relatively moderate consumption of opioids compared to neighboring nations.

It is rather unclear what the Brazilian context of opioid utilization means for the needs, practices, and outcomes related to pain care given that many countries find themselves in a major recalibration phase (e.g., with major changes in opioid utilization control and practice) as to the role of opioid-pharmacotherapy in evidence-based approaches for pain care while minimizing collateral harms [5, 22, 69, 70]. While North America and other wealthy nations have vastly increased utilization of (especially strong) opioids in a quest for ‘better’ pain care post-1990, many subsequently experience unprecedented adverse consequences from opioid-related fatalities, hospitalizations, and dependence, driven by persistent increases in opioid availability [11, 71, 72]. Increasing adverse consequences following recent restrictions on prescription opioids have been related to illicit/synthetic opioid products that appear to fill ‘supply gaps’ [9, 73–75].

In Brazil, codeine remains the most common opioid analgesic prescribed, but prescriptions of stronger opioids such as oxycodone are becoming more common, while detailed dispensing information is lacking. Codeine products are mainly prescribed for acute health conditions, whereas non-opioid analgesics and NSAIDs are the most utilized drugs for pain-related conditions. While chronic pain is reported to be prevalent in municipal survey samples [36, 37, 76–78], satisfaction with pain treatment is reported by few patients [36]. In contrast, wealthy countries with higher levels of opioid usage report more patients (50 %-60 %) being satisfied with pain care [23]. Notably, the lack of satisfactory pain management in Brazil corresponds with self-medication practices involving any medicines not prescribed by a doctor in as much as 16–25 % of the general population, with non-opioid/non-prescription analgesics being the most used self-medication drugs among Brazilians engaging in self-medicating activities [79, 80]. This appears to conflict with international agreements recognizing adequate access to opioid medicines is “indispensable for the relief of pain and suffering” [81, 82]. While many wealthy countries clearly have ‘overshot’ on this principle with excessive opioid dispensing for long periods, general increases in medical opioid availability and use would appear appropriate in Brazil in order to achieve adequate pain care.

The low levels of medical opioid utilization in Brazil are noteworthy given the relatively high use of other psychotropic medication (e.g., benzodiazepines, antidepressants, amphetamines) [51, 83–85]. Considering the low opioid utilization levels in Brazil, it is likely that regulatory barriers, health policies, financial restraints, and medical/patient education play a part. Brazil features multiple restrictive regulations for opioid prescribing, including restrictive prescription formalities, low-dose limits, prescriber limitations to authorized physicians and dentists, among others, and so – even within Latin America – is considered ‘highly restricted’ for opioid access [25]. These regulations have been justified as preventing opioid non-medical use while unduly neglecting pain care and medication needs in practice [4, 22]. Other factors appear relevant, including inadequate (under-resourced) public health policies and services for the majority of the population in a two-tiered health system, poor palliative care, and lack of specialist pain treatment training, programs, and evidence-based guidelines towards improved opioid-based medical care [22, 26, 35]. Financial barriers towards procuring opioid medicines for many Brazilians have also been identified, including high cost to patients, for many of whom opioids are simply unaffordable [4, 25, 50]. Furthermore, far-reaching inadequacy of training and knowledge among health-practitioners seem to translate into systemic mis-information and -practice on appropriate opioid use, fear of diversion, abuse/dependence, or even prosecution, contributing to systemic adversity (‘opio-phobia’) to medical opioid utilization [4, 22, 86].

National surveys indicate a substantial increase in non-medical (i.e., non-prescribed) opioid use over time in Brazil. This involves only about 0.5–1.5 % prevalence and is thus substantially lower than levels reported for North America [13, 87]. These observations appear to confirm that overall population-level opioid availability determines corresponding levels of opioid-related harms (e.g., non-medical use or mortality) [69, 71, 88]. Available indicators are limited in regard to contexts of non-medical use. For instance, it is unclear why people are using prescription opioids non-medically. Additional studies are needed to investigate motives for the non-medical use of prescription opioids in Brazil. Moreover, there is a lack of information on possible sociodemographic/economic factors influencing non-medical opioid use. For example, higher prevalence of non-medical opioid use among women commonly observed might be related to higher prevalence of chronic pain [37, 79] and other (self-)medication use among women in general [40, 84, 85, 89, 90]. On the other hand, opioid-related deaths in Brazil post-2000 is twice as high among men as compared to women [67], as is also observed – albeit at higher levels – for other psychotropic drugs [68]. The influence of sociodemographic, behavioral, or economic factors towards these divergences is unclear yet should be empirically examined.

The use of illicit opioids (e.g., heroin or illicit fentanyl) appears to be highly uncommon in Brazil, both in general as well as in marginalized (e.g., street drug use) populations. This is rather different from North America or Europe, where illicit opioid drugs form a major part of the epidemiology of non-medical substance use and related harms, including mortality [2, 10, 91]. While illicit substance use (other than cannabis) in Brazil has traditionally centered around psychostimulants (e.g., cocaine/crack-cocaine, amphetamines) and non-injection drug use [51, 58, 66, 92], questions about the principal drivers of this profile are worthwhile but empirically unanswered. Brazil is not part of the major global locations or paths of illicit opioid production and supply [3]. Notably, recent sporadic heroin use has been linked with local pockets of international migration in urban settings [64]. Yet overall, it is unclear whether the persistently low availability/use of opioids in the Brazilian context has shaped a scenario of low illicit opioid use (e.g., through limited general exposure, pathways from medical to non-medical/illicit use, diversion potential) or whether these ought to be considered more independent phenomena.

## Conclusions

As sketched out by this overview, there is only limited and rather insufficient, and especially systematic and rigorous (e.g., from peer-reviewed studies) surveillance and analytical data on opioid use, policy/practice, and harms (e.g., morbidity/mortality) in Brazil. Essential data and outcomes are widely lacking, with largely only sporadic or local indicators available while others are entirely absent. This may not be surprising for a developing/threshold country like Brazil and is consistent with common limitations for substance use and outcome indicators internationally observed elsewhere [93], although other areas of health behavior, outcomes, or systems are much better documented. Within available publications or sources, potential publication biases (e.g., underestimates due to methodological bias, lack of sample representativeness, etc.) need to be considered that could impact results and, consequently, have implications for public health and policy. Therefore, since existing and acute (e.g., pain-related) needs for improved opioid utilization and practice appear to be substantiated, better indicators for opioid use, practice, policy, and harms are clearly required. Meanwhile, Brazil – despite or because of its comparably low levels of documented opioid use and related harms – remains a worthwhile case study within the regional contexts of Latin America, as well as in contrast with the extremes of the North American ‘opioid crises’.

## Data Availability

Not applicable.

## Abbreviations

INCB: International Narcotics Control Board
S-DDD: defined daily doses for statistical purposes
NSAIDs: non-steroidal anti-inflammatory drug
ANVISA: National Health Surveillance Agency (*Agência Nacional de Vigilância Sanitária)*
ASSIST-WHO: World Health Organization Alcohol, Smoking, and Substance Involvement Screening Test
SIM: Mortality Information System (Sistema de Informações sobre Mortalidade)
